# Integrated fate assessment of aromatic amines in aerobic sewage treatment plants

**DOI:** 10.1007/s10661-020-8111-y

**Published:** 2020-04-11

**Authors:** Lin Jun Zhou, Zhi Yi Rong, Wen Gu, De Ling Fan, Ji Ning Liu, Li Li Shi, Yan Hua Xu, Zhi Ying Liu

**Affiliations:** 1 College of Biotechnology and Pharmaceutical Engineering, Nanjing Tech University, Nanjing, China; 2 Ministry of Ecology and Environment, Nanjing Institute of Environmental Sciences, Nanjing, China; 3 Lubrizol Corporation, Wickliffe, OH USA

**Keywords:** Aromatic amine, Removal rate, Biodegradation, STP, Model

## Abstract

The fate and exposure of chemicals in sewage treatment plants (STPs) are major considerations in risk assessment and environmental regulation. The biodegradability and removal of seven aromatic amines were systematically evaluated using a three-tiered integrated method: a standard ready biodegradability test, an aerobic sewage treatment simulation method, and model prediction. In tier 1, the seven aromatic amines were not readily biodegraded after 28 days. In adapted aerobic active sludge, 4-isopropyl aniline, 2,4-diaminotoluene, and 4-nitroaniline among them exhibited the degradation half-life time less than 20 h, the other four aromatic amines exhibited persistent with degradation half-life of > 60 h. In tier 2 of the aerobic sewage treatment simulation testing, 2,4-diaminotoluene, 4-nitroaniline, and 4-isopropylaniline demonstrated moderately to high overall removal. Hydraulic retention time (HRT) affects the removal with the optimum HRT was determined to be 12 h to 24. 2,6-Dimethyl aniline, 2-chloro-4-nitroaniline, 2,6-diethylaniline, and 3,4-dichloroaniline were not removed during the test, indicting these four aromatic amines will enter surface water and hence pose a potential risk to aquatic ecology. Considering the lack of an STP model in China for regulation purposes, in tier 3, we developed a Chinese STP (aerobic) (abbreviated as C-STP(O)) model that reflects a universal scenario for China to predict the fate. The predicted degradation, volatilization, and absorption showed a close relationship to the physicochemical properties of the chemicals, and had same tendency with tier 2 simulation test. The prediction showed that biodegradation rather than absorption or volatilization was the main removal process of aromatic amines in aerobic STP. With the combination of modified kinetics test with C-STP (O) model, the chemical fate can be more accurately predicted than using only the readily biodegradation result.

## Introduction

The demand for chemicals, which are either incorporated into various consumer products or accumulate in the environment, has continuously increased in recent decades. Chemical regulations such as the European Registration, Evaluation, and Authorization of Chemicals (REACH) legislation and the Toxic Substances Control Act (TSCA) established by the US EPA have been implemented to control a large number of chemicals and prevent environmental and health risks. As the largest producer and user of chemicals, China has enacted the Regulations on the Safe Management of Hazardous Chemicals to assess and manage the risks of hazardous substances.

Aromatic amines are important intermediates formed during the production of various synthetic organic chemicals and polymers, including polyurethanes, rubber additives, dyes, pharmaceuticals, pesticides, and herbicides (Zhu et al. 2011). Aromatic amines have attracted attention in recent years because of the toxicity associated with the wide distribution of these chemicals in the environment (Bruschweiler and Merlot 2017; Jurado-Sanchez et al. 2012; Muz et al. 2017a, b; Bouknana et al. 2019; Men et al. 2011). Aromatic amines are powerful carcinogens, mutagens, and hemotoxicants in humans, animals, and plants (Muz et al. 2017a, b; Slavov et al. 2018; de los Santos et al. 2015). Wastewater contains aromatic amines that have toxic effects on aquatic organisms, including fish, algae, and other aquatic fauna (Burkhardt-Holm et al. 1999; Gosetti et al. 2010; Muz et al. 2017a, b; Furuhama et al. 2015). 3,4-Dichloroaniline has been recognized as an endocrine disrupting chemicals (Tasca and Fletcher 2018). Therefore, the environmental risk of aromatic amines must be assessed to take appropriate management measures.

Environmental risk assessment (ERA) is widely used to predict the environmental hazards and exposure of released chemicals (Maltby 2006; Neves and Mol 2019). Assessment of the fate and exposure of chemicals is a basic process in ERA that aims to predict the concentration and distribution of chemicals in different environmental compartments. Sewage treatment plants (STPs) are the major secondary sources of pollution and are the focus of ERA, as chemicals may not be completely removed or degraded by chemical, physical, and biological treatment processes within STPs(Archana et al. 2017; Neves and Mol 2019; Salaudeen et al. 2018). Many aromatic amines pass through STPs owing to their persistence or continuous release (Luo et al. 2014; Tas and Pavlostathis 2014; Jurado-Sanchez et al. 2012). Numerous aromatic amines cannot be removed from STPs due to their non-biodegradability and toxicity to microorganisms and hence pose a potential risk to ecosystems (Ning et al. 2015). However, key data on the environmental fate and exposure of aromatic amines, especially data on biodegradability, are often incomplete, which slows the progress of risk assessment of aromatic amines.

Organization for Economic Co-operation and Development (OECD) has developed a tiered biodegradability testing strategy to assess the fate of chemicals (OECD 2009, 1992, 2001). First, aerobic biodegradability should be examined in a screening test for ready biodegradability. If a negative result (no biodegradation) is observed in a ready biodegradability test, the biodegradation of the chemical must be examined in a simulation test to obtain data to assess the biodegradation rate constant (*k*) in the environment or in a biological STP. A precise *k* is needed, as *k* is a very important parameter used in STP models and environmental multimedia models to predict the exposure concentration. The OECD laboratory simulation test 303 A (OECD 2001) for aerobic sewage treatment can only test the total removal rate and residual percentage in the effluent and cannot determine the fraction released to the air and sludge, while the removal rate, including absorption to surplus sludge and evaporation to the air, is critical for ERA to calculate the predicted environmental concentrations in the air and soil. Several complementary mathematical models can predict the degradative, absorptive, and evaporative fate of chemicals in STPs (Prata et al. 2018; Franco et al. 2013). These models, such as SimpleTreat (Franco et al. 2013), have been used as standard models in the European Union (EU) and in the USA to estimate the exposure in the STP and the environmental compartment.

China currently lacks an STP model for the risk assessment of produced or imported chemicals, including aromatic amines. SimpleTreat and other related models may underestimate or overestimate the risk of chemicals because the environmental conditions and scenario parameters used in China significantly differ from those in the EU and USA. An STP model based on universal scenario parameters in China must be developed to support the risk assessment of chemicals in China (MEP 2011).

In the present study, biodegradability and removal of seven aromatic amines were evaluated using a three-tiered test: a standard ready biodegradability test, an aerobic sewage treatment simulation method, and model prediction. In tier 1, a standard ready biodegradability test was performed for seven aromatic amines, and the biodegradation kinetics and *k* values in adapted active sludge were researched by a modified method. In tier 2 and tier 3, an aerobic sewage treatment simulation method and a C-STP(O) model that reflects a universal scenario for China were also developed to predict the fate of aromatic amines in STPs. Collectively, these integrated methods will help to assess the environmental risk and persistency (for persistent, bioaccumulative, and toxic (PBT)/very persistent and very bioaccumulative (vPvB) assessment) of aromatic amines more systematic.

## Materials and methods

### Standards and reagents

The following seven aromatic amines were used: 4-nitroaniline, 2-chloro-4-nitroaniline, 2,6-dimethyl aniline, 3,4-dichloroaniline, 2,6-diethylaniline, 4-isopropylaniline, and 2,4-diaminotoluene. These chemicals were all analytically pure and purchased from J&K of Beijing. Methanol (grade of high performance liquid chromatography) was purchased from Merck KGaA.

**Fig. 1:**
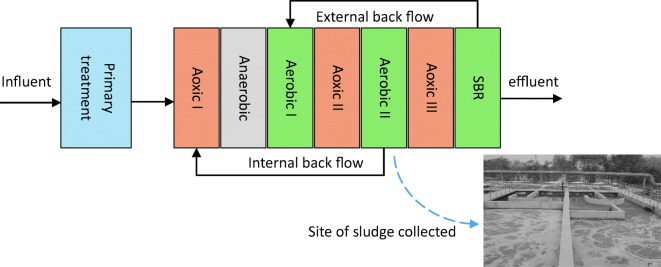
STP process where sludge was collected

Active sludge as inoculum was collected from the aerobic tank of the Nanjing Eastern STP, located at No.1, Yuanyi Road, Gaoqiao Village, Nanjing, Jiangsu province, China. This STP treat domestic sewage with daily capacity of 350,000 m^3^. The major treat process of this STP is anaerobic-anoxic-aerobic (A^2^/O) (Fig. 1).

### Ready biodegradability test (unadapted aerobic active sludge testing)

The ready biodegradability test was based on the manometric respirometry method of the OECD test guideline 301F (OECD 1992). Tests were conducted in 500 mL closed bottles. Mineral medium (297 mL), active sludge suspension liquid (3 g L^−1^, 3 mL), and the aromatic amine (~ 9 mg) were added to a bottle, yielding sludge and aromatic amine concentrations of approximately 30 mg L^−1^. A bottle without test substances served as a blank, and a bottle containing sodium benzoate served as a reference control. The bottles were stirred in a closed flask at a constant temperature (22 ± 2 °C) for 28 days. Oxygen consumption within 28 days was measured using a biochemical oxygen demand (BOD) analyzer (Lovibond, Germany). The biodegradation rate *R* of the aromatic amines was calculated as.

1 $$ R=\frac{Q_T-{Q}_C}{C_T\times {ThOD}_T}\times 100 $$

where *Q*_*T*_ and *Q*_*C*_ refer to the oxygen consumption of the test substance and blank, respectively (mg L^−1^), *C*_*T*_ is the concentration of the test substance (mg L^−1^), and *ThOD*_*T*_ is the theoretical oxygen demand of the test substance (mg_O2_·mg^−1^).

### Kinetic test of aromatic amines (adapted aerobic active sludge testing)

Sludge with an mixed liquor suspended solids (MLSS) concentration of approximately 3 g L^−1^ which have been adapted to the aromatic amines was collected from the aerobic tank, transferred to a conical flask, and then stirred continuously at 500 r/min to ensure that the dissolved oxygen (DO) concentration exceeded 2 mg L^−1^. Aromatic amines were added to the flask to yield an initial concentration of 1 mg L^−1^ and then incubated for degradation. The residual concentrations of the test substances in the solution were determined at regular intervals during a 36-h period via HPLC-MS/MS. A first-order rate constant was determined using simple linear regression (Nyholm et al. 1996). First-order kinetics was defined as

2 $$ d={d}_0{e}^{- kt} $$

where *d* is the residual rate of the test substance at time *t* (%), *d*_*0*_ is the initial percentage of the test substance (%), *k* is the rate constant (h^−1^), and *t* is time (*h*). The degradation half-life (*h*) DT_50_ was calculated as.

3 $$ {\mathrm{DT}}_{50}=\mathrm{ln}2/k $$

### Simulation test in an aerobic sewage treatment system

The simulation test in an aerobic sewage treatment system was based on method 303A of the OECD test guidelines (OECD 2001). This system consists of an aerobic tank with an operating volume of 4.8 L and a secondary settling tank with a volume of approximately 2.5 L. The aerobic tank contained 3 g L^−1^ MLSS and air to maintain sufficient dissolved oxygen.

Trace elements in synthetic wastewater were added to the raw water at a ratio of 1:100 (v/v) to yield final concentrations of 110 mg L^−1^ yeast extract, 160 mg L^−1^ peptone, 30 mg L^−1^ urea, 28 mg L^−1^ K_2_HPO_4_, 7 mg L^−1^ NaCl, 4 mg L^−1^ CaCl_2_, and 2 mg L^−1^ MgSO_4·_7H_2_O. Synthetic wastewater was used to provide nutrition to microorganisms.

The raw water containing synthetic sewage flowed into the aerobic tank via a continuous pump. Approximately 1/8 of the volume of the active sludge was removed daily from the aeration vessel to maintain a sludge retention time of 8 days and an MLSS level of approximately 3 g L^−1^. Hydraulic retention time (HRT) is a critical design parameter for system operations. This parameter was controlled by changing the flow rate of water. The specific operating parameters of the aerobic sewage treatment system are listed in Table 1.

**Table 1:** Operating parameters of the aerobic sewage treatment system

Definition	Value	Unit
Volume of raw water	35	L
Volume of aerobic tank	4.8	L
Volume of secondary settling tank	2.2	L
Mixed liquor suspended solids of aerobic tank	3	g L^−1^
Flow rate of lifting pump	6.7	mL min^−1^
Flow rate of returned sludge pump	30	mL min^−1^
Hydraulic retention time	12, 24, 6	h
Sludge retention time	9.2	day
Influent total dissolved organic carbon	100	mg·L^−1^
Temperature	22 ± 2	°C

Under stable system operation, the removal rate of dissolved organic carbon (DOC) reached 80%; at this point, 1 mg L^−1^ aromatic amines was added into the raw water.

Raw water, active sludge, and effluent water from the system were periodically analyzed for pH, DO, and oxidation reduction potential (ORP) by using a portable multi-parameter analyzer (HACH HQ40d, America). The MLSS in the aerobic tank and the SS in the effluent water were determined using a gravimetric method.

A 5-mL volume was sampled from the raw water and the secondary settling tank, filtered using a 0.45-μm filter membrane, and then acidized with 0.1 mol L^−1^ hydrochloric acid. The DOC removal rate in the sample was determined using a total organic carbon (TOC) analyzer (Jena, Germany). The specific DOC removal rate was calculated as

4 $$ {D}_t=\frac{c_i-{c}_e}{c_i}\times 100 $$

where *D*_*t*_ is the specific DOC removal rate (%), *c*_*i*_ is the concentration of DOC in the influent water (mg·L^−1^), and *c*_*e*_ is the concentration of DOC in the effluent water (mg L^−1^).

Influent and effluent samples were analyzed to determine the concentration and removal of aromatic amines. A 5-mL sample was mixed with 5 mL of methanol for 5 min of ultrasonic extraction. The mixture was filtered using a 0.22-μm filter membrane. The filtrate was then analyzed via high-performance liquid chromatography–mass/mass spectrometry (HPLC–MS/MS) with an Agilent 1290 HPLC system and an AB SCIEX Triple Quad 4500 MS/MS detector. A ZORBAX Eclipse plus C18 column (2.1 mm × 150 mm, 3.5 μm; Agilent, USA) was used for the separation. The column temperature was set at 30 °C, the flow rate was set at 0.5 mL min^−1^, and the injection volume was set at 2 μL. Mobile phase A contained 2 mmol L^−1^ ammonium formic acid in ultrapure water, and mobile phase B contained acetonitrile. The solvents were mixed as follows: 0.01 min 95% A, 5% B; 6 min 5% A, 95% B; 6.1 min 95% A, 5% B; and 7 min 95% A; 5% B. In this method, all target analysis substances were detected in negative electrospray ionization (ESI^−^) mode. The optimum MS parameters included parent ions, product ions, collision energy, de-clustering potential, entrance potential, and collision cell exit potential. The detector conditions were as follows: 35000 Pa curtain gas, 4500 V ion spray voltage, and 500 °C ion source temperature.

The removal rate of aromatic amines was calculated as

5 $$ R=\frac{ST_i-{ST}_t}{ST_i}\times 100 $$

where *R* is the specific removal rate of the test substance (%), *ST*_*i*_ is the concentration of the test substance in the influent water (mg L^−1^), and *ST*_*e*_ is the concentration of the test substance in the effluent water (mg L^−1^).

### Construction of the STP model

#### Basic concept of C-STP(O)

**Fig. 2:**
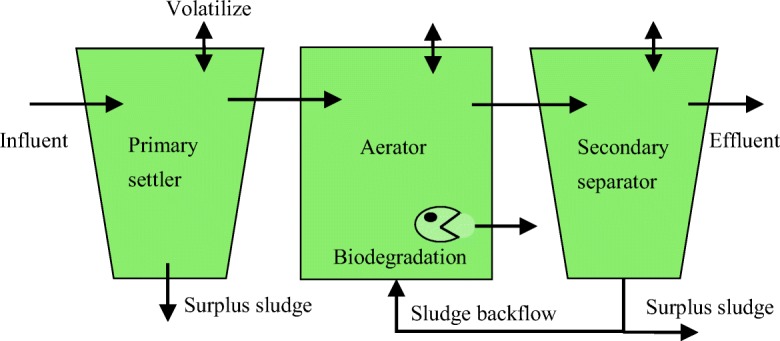
Conceptual diagram of the C-STP(O)

The calculation process of C-STP(O) was similar as SimpleTreat model (Franco et al. 2013). The Level III fugacity-based C-STP(O) model can provide a steady-state distribution of a chemical in a three-stage STP consisting of a primary settling tank, an active sludge-based aeration tank, and a secondary settling tank. Figure 2 illustrates the model concept of C-STP(O).

A mass balance can be written for each tank as follows:

6 $$ -k{c}_i{V}_i+\sum \left[A\left(i,j\right)\times {c}_i\right]+\sum \left[X\left(i,j\right)\times {c}_i\right]=0 $$

where *k* is the first-order biodegradation rate constant (s^−1^) under the assumption that biodegradation occurred only in aerator, *c*_i_ is the concentration in tank *i* (mol m^−3^), *V*_i_ is the volume of tank *i*, *A*(*i*,*j*) is the flow rate of a medium irreversibly flowing from tank *i* to *j* (m^3^ s^−1^), and *X*(*i*,*j*) is the volume flow rate for diffusive transport from tank *i* to *j*(m^3^ s^−1^).

*V*_i_, *A*(*i*,*j*), and *X*(*i*,*j*) were derived from the base properties of the modeled chemicals and parameters specific to the STP. *c*_i_ was obtained from multiple mass balance calculations and was the standard output used to calculate the removal and distribution in each environmental medium. In general, the model was constructed based on the input physicochemical parameters of the chemicals and the process parameters of the STP. Three important physicochemical properties, partition coefficient of organic carbon/water (lg*K*_oc_), Henry’s constant (*H*) and *k*, may be obtained from laboratory test data or from the literature. lg*K*_oc_ is used to predict the absorption to sludge, *H* is used to calculate the evaporation to the air, and *k* is used to calculate the biodegradation in the aerator tank.

#### Development of the C-STP(O) model

The scenario parameters used in the SimpleTreat model are mainly based on the EU, and several scenario parameters may markedly affect the results. The direct use of the SimpleTreat model to estimate the fate of chemicals may overestimate or underestimate exposure in water ecosystems in China. Thus, the environmental conditions in China and the water parameters of STPs were investigated using published studies and tests, and the data were used to develop the C-STP(O) model on the basis of the SimpleTreat model.

The average environmental temperature (*T*) in 2014 was 10 °C on the basis of the *China Climate Bulletin* (CMA-China 2014). *T* affects not only volatilization but also *k*, as shown in the following equation (Franco et al. 2013):

7 $$ k={k}_{T1}\times {1.072}^{T-{T}_1} $$

where *k*_T1_ is the test or reference biodegradation rate and *T*_1_ is the temperature at which *k*_T1_ is obtained.

The wind speed was set to 2 m s^−1^ but ranged from 1 to 2 m s^−1^ in many cities. Temperature and wind speed can affect the volatilization of chemicals, and temperature was used to calibrate water solubility and vapor pressure by using the Clapeyron–Clausius equation.

In the SimpleTreat model, the volume of wastewater (*Q*, m^3^·pe^−1^·d^−1^), biochemical oxygen demand (BOD_5_, mg·pe^−1^·L^−1^), and concentration of suspended solid (SS, mg·pe^−1^·L^−1^) are averaged on a per-person basis; that is, the values are calculated by the number of inhabitants (NI, pe) multiplied by the volume or the amount per person. Considering that many plants treat both domestic wastewater and industrial wastewater and that the capacity of STPs is larger in China than in the EU, we used the total volume of wastewater (*Q*, m^3^·d^−1^), BOD_5_ (mg L^−1^), and SS (mg L^−1^) to modify the model. The average *Q* of the STP was set to 35,000 m^3^ d^−1^ on the basis of the statistical data for all 4436 STPs in the “Announcement of the Publication of the Operational National Urban Sewage Treatment Facilities List for 2014” published by the Ministry of Environmental Protection (MEP) of China (MEP-China 2014).

**Fig. 3:**
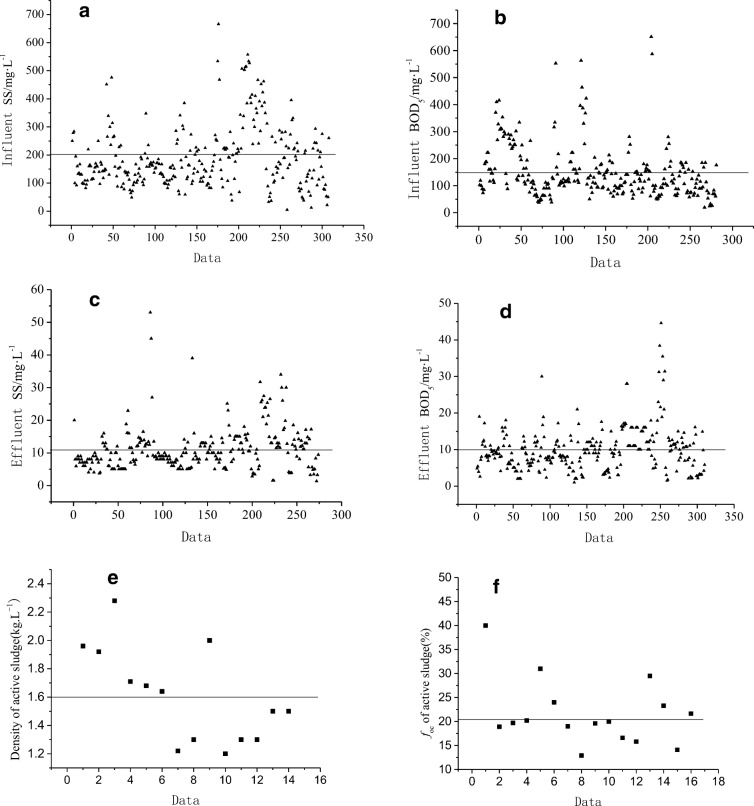
Water quality and active sludge parameter of STP in China

Water quality and sludge properties (e.g., BOD_5_ and SS of influent and effluent; density and organic carbon content of sludge) can affect the absorption behavior of chemicals. Thus, a series of data was collected to reflect Chinese characteristics. The average influent SS (Fig. 3a), influent BOD_5_ (Fig. 3b), effluent SS (Fig. 3c), and effluent BOD_5_ (Fig. 3d) were 200, 150, 12, and 10 mg L^−1^, respectively, as calculated from 308, 281, 274, and 310 data sets. The water quality of STP effluent discharged to surface water meets standard B at the first level specified in the Chinese “Discharge Standard of Pollutants for Municipal Wastewater Treatment Plant” (SEPA-China 2002), in which neither BOD_5_ nor SS should exceed 20 mg L^−1^. When discharged into surface water, the chemicals absorbed in SS pose a potential risk to aquatic ecosystems. Thus, the BOD_5_ and SS of the effluent were set to 20 mg L^−1^ in the model to limit risk.

The HRTs of the primary settler, aerator, and solid/liquid settler were set to 2, 10, and 4 h, respectively, on the basis of the Chinese “Code for Design of Outdoor Wastewater Engineering” standard (MC-China 2006).

The density and organic carbon content of active sludge at 10 sites in China, including Beijing, Nanjing, Shanghai, and Shenyang, were analyzed. The density of the active sludge (Fig. 3e) was 1.2–2 kg L^−1^, with a mean of 1.6 kg L^−1^, and the organic carbon content of the active sludge (Fig. 3f) was 18–31%, with a mean of 20%. These two average values were used in the model.

Table 2 compares the environmental conditions and scenarios for China and the EU in SimpleTreat. The concentrations of BOD_5_ and SS were lower in Chinese raw water than in EU raw water.

**Table 2:** Comparison of environmental conditions and scenarios in SimpleTreat for China and the EU

Parameter	SimpleTreat^(1)^ Value	C-STP(O)	
		Value	References
Temperature (K)	288	283	CMA-China 201
Wind Speed (m s^−1^)	3	2	
Sewage Flow (m^3^ d^−1^)	2000	35,000	MEP-China 2014
HRT of primary settler (h)	2	2	MC-China 2006
HRT of aerator (h)	6.9	10	
HRT of SLS (h)	6	4	
SS of influent (mg/L)	450	200	Fig.2a
SS of effluent (mg/L)	30	20	SEPA-China 2002
BOD_5_ of influent (mg/L)	270	150	Fig.2b
BOD_5_ of effluent (mg/L)	28	20	SEPA-China 2002
Density of active sludge (kg·L^−3^)	1.3	1.6	Fig.2f
Organic carbon content of active sludge	0.37	0.2	Fig.2e

^(1)^Franco et al. 2013

## Result and discussion

### Biodegradability and kinetics of aromatic amines

#### Ready biodegradability

The ready biodegradation data obtained for aromatic amines in the standard OECD manometric respirometry test are listed in Table 3. No biodegradation of the aromatic amines occurred, as indicated by the − 9.8–9.9% biodegradation rate after 28 days. Failure to achieve biodegradation in the ready biodegradability test can be attributed to many factors, such as toxic effects on microorganisms when working at high test concentrations (30 mg/L). The ready biodegradation test is very stringent: unadapted microorganisms that are present in low concentrations and with low diversity have limited opportunities for adaptation to and biodegradation of the tested chemical (test duration 28 days).

**Table 3:** Biodegradation and kinetic parameters of seven aromatic amines

Chemical	Ready Biodegradability		Biodegradation kinetics in adapted active sludge			
	Degradation rate/%	*k*/h^−1^	First-order kinetic equation (d)	Correlation coefficient (*r*^2^)	*k*/h^−1^	DT_50_/h
2,6-Dimethyl aniline	9.9	0	0.9265e^-0.011*k*^	0.9422	0.011	63.0
4-Nitroaniline	2.5	0	0.8482e^-0.0410*k*^	0.9847	0.041	16.9
2-Chloro-4-nitroaniline	0	0	0.997e^-0.0056*k*^	0.9597	0.006	124
4-Isopropylaniline	0	0	0.94378e^-0.1154*k*^	0.9422	0.115	6.01
2,6-Diethylaniline	− 3.4	0	1.0705e^-0.0031*k*^	0.9943	0.003	224
2,4-Diaminotoluene	− 1.6	0	0.8002e^-0.0420*k*^	0.9479	0.042	16.5
3,4-Dichloroaniline	2.3	0	0.9576e-^0.0050*k*^	0.8456	0.005	139

Thus, *k* was assumed to be 0 for all aromatic amines, as suggested by the EU model.

A negative result in the ready biodegradability test does not necessarily indicate that the chemical cannot be degraded under relevant environmental conditions, but it can be considered as an indication of a potentially persistent chemical and may trigger the next level of testing, i.e., either a simulation test or an inherent biodegradability test.

#### Biodegradation rate (k) and kinetics in adapted aerobic active sludge

**Fig. 4:**
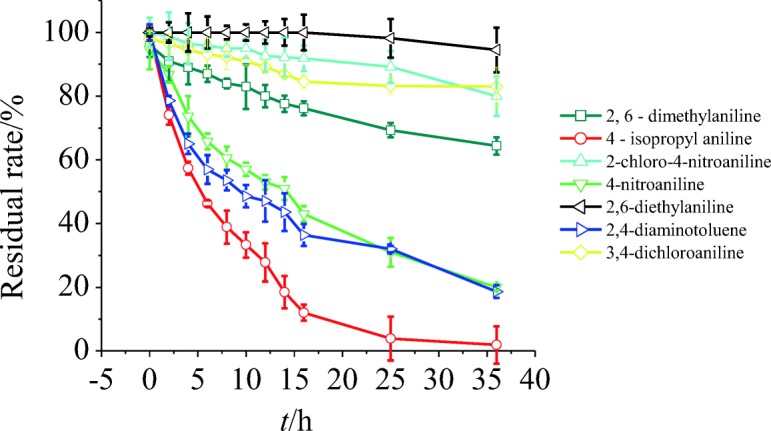
Residual rates of aromatic amines over time in kinetic test

To accurately predict the exposure and removal of chemicals in an STP, the biodegradation rate and kinetics in the aerobic active sludge need to be tested as input rather than using the exploration criteria from screening tests, as suggested by the EU model. The residual rates of aromatic amines over time are shown in Fig. 4, and the first-order kinetic equation is presented in Table 3. Aromatic amines were degraded at different rates during the biodegradation kinetic test in the adopted aerobic active sludge. The correlation coefficients of first-order kinetic curve fitting exceeded 0.9, except that for 3,4-dichloroaniline (*r*^2^ = 0.8456). This result demonstrates that all of the aromatic amines were degraded according to first-order kinetics. The *k* values of 4-isopropylaniline, 4-nitroaniline, and 2,4-diaminotoluene were 0.115, 0.041, and 0.042 h^−1^, respectively, which corresponded to DT_50_ values of 6.01, 16.9, and 16.5 h. The four other anilines were relatively persistent, with DT_50_ > 60 h.

Substituted groups significantly affect chemical biodegradation. Quantitative structure–activity relationships (Cuissart et al. 2002; Byrns 2001) show that halogens, nitro groups, and quaternary carbons can negatively influence the biodegradation of aromatic chemicals because these groups can reduce the electron cloud density and exhibit a strong electron-withdrawing ability. Therefore, nitro groups and chlorine atoms can inhibit biodegradation.

Three aromatic amines were efficiently degraded in the adapted active sludge; these results contradicted those of the ready biodegradability test, possibly because of the reduced concentration of test substances, the increased concentration of the inoculum, and the adapted aerobic active sludge. An increased biomass concentration may allow more bacteria to adapt to chemical degradation (Ahtiainen et al. 2003). Jiang et al. (2019) found that the main functional genera such as *Proteobacteria*, *Achromobacter*, *Defluviimonas*, *Enterobacter*, *Pseudomonas*, and *Pseudoxanthomonas*, were detected in the system and were found to be responsible for reduction of anilines. Hou et al. (2018) found that the proportions of *Pseudomonas*, *Thermomonas*, and *Acinetobacter* in biofilters increased significantly and several new bacterial taxa appeared after aniline acclimation. Cui et al. (2017) showed that the acclimated mixed culture presented better aniline degradation abilities was mainly composed of *Serratia*, *Escherichia*/*Shigella*, *Bacillus*, and *Acinetobacter*. This is also consistent with Kausar et al.’s (2016) research results in which they found that some isolated bacteria can capable of degrading > 75% of 4-nitroaniline after 24 h under aerobic conditions.

In addition, biodegradation kinetics and removal are dependent on the initial aniline concentration, temperature, pH, and substrate (Yang et al. 2019; Huang et al. 2018).

### Removal in a simulated aerobic sewage treatment system

#### Performance of the aerobic sewage treatment system

**Fig. 5:**
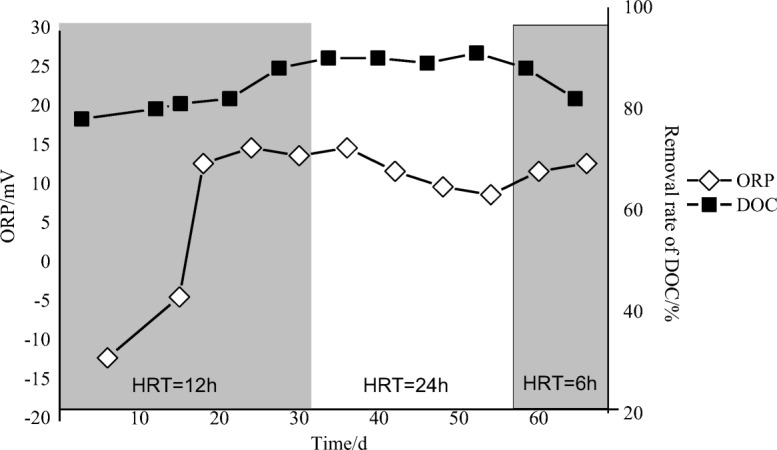
Conditions and performance of the aeration tank in the aerobic sewage treatment system

The aerobic sewage treatment system was operated at a constant 22 °C in the laboratory. The pH of the aeration tank ranged from 6.9 to 8.3, and the DO ranged from 4.39 to 8.68 mg L^−1^, indicating aerobic conditions. Figure 5 shows the ORP changes over time in the aeration tank. During the first 20 days of the test, the ORP increased from − 11.9 to 15 mV in the aerobic tank and then remained at an average value of 11 mV. A previous study reported that ORP values are strongly correlated with DOC removal from aeration tanks and that DO and temperature affect redox status and pollutant removal efficiency (Li and Bishop 2004). The ORP increase implied that the active sludge was in a relatively high oxidizing status during domestication.

During the study phase, the system was operated at three different HRTs: 12, 24, and 6 h. Consequently, the total experiment may be divided into three periods; the initial DOC removal efficiency was 80% during the first 30 days (HRT = 12 h); the efficiency was maintained at 90% from 31 to 60 days (HRT = 24 h) but decreased during the last 10 days (HRT = 6 h). The experiment demonstrated that the HRT in the second period could store sufficient biomass to continuously remove approximately 90% of the DOC from the aerobic tank (Ghyoot and Verstraete 2000). The HRT must not be excessively short, to avoid the washout of dispersed bacteria, and must not be excessively long, to prevent the formation of bacterial aggregates as well as the growth of higher organisms grazing on bacteria (Li and Bishop 2004).

#### Influence of HRT on the removal of aromatic amines

**Fig. 6:**
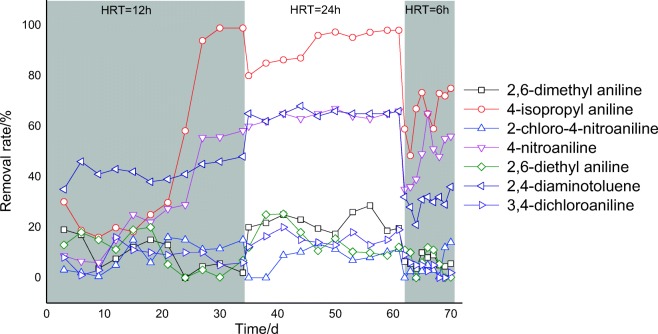
Remove efficient over time of aromatic amines in stimulate test

After acclimatization of the active sludge, the HRT was controlled to observe its effect on the removal of aromatic amines. The removal rate varied with different HRTs, as shown in Fig. 6 and Table 4. At HRT = 12 h, the removal rates of all seven chemicals were extremely low at the start of the 20-day period. An exception was 2,4-diaminotoluene, with a removal rate of approximately 45%. The removal rates of 4-isopropylaniline and 4-nitroaniline gradually increased on day 20 and reached approximately 100% and 58% on day 35, respectively. Stagnation and degradation for approximately 15 days were observed for these two chemicals. Microorganisms may need to adapt to chemicals, but degradation-competent bacteria may also grow during this period.

**Table 4:** Tested average total removal rate (%) in OECD 303A stimulate test

Aromatic amines	HRT = 24 h	HRT = 12 h	HRT = 6 h
2,6-Dimethyl aniline	20	9.0	6.4
4-Nitroaniline	63	57^(1)^	48
2-Chloro-4-nitroaniline	11	9.1	4.4
4-Isopropyl aniline	95	97^(1)^	66
2,6-Diethyl aniline	12	10	6.2
2,4-Diaminotoluene	64	42	30
3,4-Dichloroaniline	19	8.1	3.8

^(1)^Calculated by plateau phase data

From day 31 to day 65 (HRT = 24 h), the average removal rates of 2,4-diaminotoluene, 4-nitroaniline, and 4-isopropylaniline were 65%, 64%, and 95%, respectively. The remaining four chemicals were not effectively removed. In the last 10 days (HRT = 6 h), the removal rates of three chemicals decreased, with average values of 48%, 59%, and 30% for 4-nitroaniline, 4-isopropylaniline, and 2-chloro-4-nitroaniline, respectively. However, the rates became unstable. The optimum HRT was determined to be 12 to 24 h under the set conditions. Boonnorat et al. (2019) also found that HRT plays a crucial part in micropollutant biodegradation of bio-augmented active sludge system, too short, an HRT (12 h) results in low micropollutant removal efficiency, and too long, an HRT (24 h) contributes to low daily throughput and high treatment operation cost. As a result, and moderate HRT (18 h) is operationally and economically optimal for bioaugmented-activated sludge system treating low-micropollutants wastewater.

The other four chemicals showed no removal during the entire test period. The poor removal rate indicated that aromatic amines may not be adequately removed during biological wastewater treatment and hence pose a potential risk to aquatic ecology. Tasca and Fletcher (2018) reviewed that many chloroanilines persist in environment for years. 3,4-Dichloroaniline also had high persistence in sediment-water systems (Yuan et al. 2017). However, their removal rate at anaerobic sludge need to be assessed as some evidences shown they may be biodegraded at anaerobic conditions (Duc 2019).

### Prediction of the STP model

The testing conditions in the simulated aerobic sewage treatment system differed from those in the universal scenario for China. In particular, the SS in the influent water was 0, and the optimum HRT ranged from 12 to 24 h in the test, while the SS was 200 mg L^−1^ and the HRT was 10 h in the C-STP(O) model. Thus, the fate of the seven aromatic amines in the STP was predicted using the C-STP(O) model to determine the overall removal efficiency of absorption, volatilization, and biodegradation. The results are representative of a universal scenario for China. Table 5 shows the predicted mass flows of the seven chemicals obtained through the C-STP(O) model.

**Table 5:** Predicted and tested removal rates by C-STP(O)

Substance	lg*K*_oc_	*H/*Pa·m^3^·mol^−1^	Predicted removal rate/%							
			Using *k* from ready biodegradability				Using *k* from adapted active sludge			
			Absorption	Volatilization	Degradation	Total	Absorption	Volatilization	Degradation	Total
2,6-Dimethyl aniline	2.54	3.49	1.61	6.49	0	8.10	1.59	6.59	9.3	17.5
4-Nitroaniline	1.64	0.035	0.21	0.08	0	0.29	0.19	0.06	29.0	29.3
2-Chloro-4-nitroaniline	2.25	9.67 × 10^−4^	0.85	0	0	0.85	0.83	0	5.61	6.44
4-Isopropylaniline	2.53	0.035	1.62	0.07	0	1.69	1.21	0.04	52.9	54.1
2,6-Diethylaniline	2.65	0.0112	2.12	0.02	0	2.15	2.09	0.02	2.85	4.96
2,4-Diaminotoluene	0.96	9.52 × 10^−5^	0.04	0	0	0.04	0.04	0	29.6	29.6
3,4-Dichloroaniline	2.05	2.30	0.53	4.52	0	5.05	0.52	4.33	4.58	9.43

Using *k* = 0 from ready biodegradability test as an input of the C-STP(O) model to calculate the removal efficiency, we predicted low removal efficiencies for all aromatic amines (Table 5). This removal was too conservative to reflect the real fate in STPs and in environmental compartment. Using modified *k* from adapted aerobic active sludge as input, C-STP(O) model calculate the higher removal efficiency. The total removal of 2,6-dimethyl aniline, 2-chloro-4-nitroaniline, 2,6-diethylaniline, and 3,4-dichloroaniline were ranging from 4.96 to 17.5%. These results are close to 8.1~10% obtained in simulation test at an HRT of 12 h. The other three partially biodegradable aromatic amines: 4-isopropylaniline, 2,4-diaminotoluene and 4-nitroaniline achieved total removal of 54.1%, 29.6%, and 29.3%, respectively, much closer to 97%, 42%, and 57% obtained in simulation test at a HRT of 12 h. These predicted results are also conservative to meet regulations requirement. So, with the combination of modified kinetics test with C-STP (O) model, the chemical exposure can be more accurately and safely predicted than using only the readily biodegradation result.

The results also showed a close relationship to the physicochemical properties of the chemicals. Of the seven aromatic amines, 3,4-dichloroaniline, and 2,6-dimethyl aniline showed slight volatilization, with volatilization removal rates of 4.33% and 6.59%, respectively. Whitman’s two-film theory (Mackay 2001) states that the overall mass transfer coefficient for a chemical is defined in terms of the gas- and liquid-phase “single-film” mass transfer coefficient. The gas- and liquid-phase mass transfer coefficients become equal when *H* = 25 Pa m^3^ mol^−1^; chemicals are less volatile when *H* = 2.5 Pa m^3^ mol^−1^, at which value the resistance of the liquid phase is 10 times greater than that of the gas phase. If *H* is less than 0.04 Pa m^3^ mol^−1^, which is the value for water, the chemical volatilization rate is lower than that of water; the chemical can be considered non-volatilized because it can be concentrated in water when volatilization occurs. The volatilization removal rates satisfied the theory in that the *H* values of 3,4-dichloroaniline and 2,6-dimethyl aniline were 2.30 and 3.49 Pa m^3^ mol^−1^, respectively. Previous study showed that volatilization of aniline itself accounts for 46.1% of the total removal at 50 °C in simulated wastewater (Yang et al. 2019).

No significant absorption was observed for the seven aromatic amines because the lg*K*_oc_ values were within 0.96–2.65, which is less than 3.3, the criterion for significant absorption (Droge and Goss 2013). This results is consistent with the study that 3,4-dichloroaniline will slightly or moderate absorb to sediments (Yuan et al. 2017).

## Conclusions

The biodegradability and removal rates of seven aromatic amines were systematically tested using a three-tiered test: a standard ready biodegradability test, an aerobic sewage treatment simulation method, and model prediction.

The seven aromatic amines were not readily biodegradable after 28 days, with biodegradation rates ranging from − 3.4 to 9.9%. The biodegradation results in aerobic active sludge adapted to the aromatic amines showed that these aromatic amines were degraded according to first-order kinetics. 4-Isopropyl aniline, 2,4-diaminotoluene, and 4-nitroaniline among them exhibited the degradation half-life time less than 20 h. 2,6-Dimethyl aniline, 2-chloro-4-nitroaniline, 2,6-diethylaniline, and 3,4-dichloroaniline exhibited persistence, with DT_50_ > 60 d.

In the simulation test of aerobic sewage treatment, 4-isopropylaniline, 2,4-diaminotoluene, and 4-nitroaniline exhibited overall removal rates of 95%, 64%, and 63% at HRT = 24 h, respectively, but were not biodegradable in the OECD ready biodegradability test. HRT significantly influenced the removal of these three aromatic anilines, with the optimum HRT was determined to be 12 to 24 h. The four other chemicals showed little removal over the test period, indicting these four chemicals will enter surface water and hence pose a potential risk to aquatic ecology.

A C-STP(O) model that reflects a universal scenario for China was also developed to predict the fate of the aromatic amines. 4-Isopropylaniline, 2,4-diaminotoluene, and 4-nitroaniline were mostly degraded in the aerobic tank with biodegradation and removal rates of 54.1%, 29.6%, and 29.3%, respectively. 3,4-Dichloroaniline and 2,6-dimethyl aniline showed slight volatilization, with volatilization removal rates of 4.33% and 6.59%, respectively. No absorption occurred for the seven anilines.

With the combination of modified kinetics test with C-STP (O) model, the chemical fate can be much accurately predicted than using only the readily biodegradation result. The results of this study will support the ERA of aromatic amines and presents the application of the model as a standard risk assessment tool in chemical environmental regulation in China.
